# Comparing the rate of immunotherapy treatment change due to toxicity by sex

**DOI:** 10.1002/cnr2.1932

**Published:** 2024-01-08

**Authors:** Kevin J. Chua, Shane Kronstedt, Alain Kaldany, Arnav Srivastava, Sai Krishnaraya Doppalapudi, Hao Liu, Ahmad A. Tarhini, Margaret Gatti‐Mays, Elizabeth Gaughan, Siwen Hu‐Lieskovan, Raid Aljumaily, Kenneth Nepple, Bryan Schneider, Joshua Sterling, Eric A. Singer

**Affiliations:** ^1^ Rutgers Cancer Institute of New Jersey and Rutgers Robert Wood Johnson Medical School Section of Urologic Oncology New Brunswick New Jersey USA; ^2^ Rutgers Robert Wood Johnson Medical School Piscataway New Jersey USA; ^3^ Department of Biostatistics and Epidemiology Rutgers School of Public Health Piscataway New Jersey USA; ^4^ Departments of Cutaneous Oncology and Immunology Moffitt Cancer Center Tampa Florida USA; ^5^ Division of Medical Oncology The Ohio State University Comprehensive Cancer Center Columbus Ohio USA; ^6^ Division of Hematology/Oncology The University of Virginia Health System Charlottesville Virginia USA; ^7^ Department of Internal Medicine Division of Oncology University of Utah School of Medicine and Huntsman Cancer Institute Salt Lake City Utah USA; ^8^ Department of Hematology/Oncology Stephenson Cancer Center University of Oklahoma Health Sciences Center Oklahoma City Oklahoma USA; ^9^ Department of Urology University of Iowa Holden Comprehensive Cancer Center Iowa City Iowa USA; ^10^ Indiana University Melvin and Bren Simon Comprehensive Cancer Center Indianapolis Indiana USA; ^11^ Division of Urologic Oncology The Ohio State University Comprehensive Cancer Center Columbus Ohio USA

**Keywords:** cancer, drug side effects, immunotherapy, oncology, sex differences

## Abstract

**Background:**

Immuno‐oncology therapy (IO) is associated with a variety of treatment‐related toxicities. However, the impact of toxicity on the treatment discontinuation rate between males and females is unknown. We hypothesized that immune‐related adverse events would lead to more frequent treatment changes in females since autoimmune diseases occur more frequently in females.

**Aims:**

Our aim was to determine if there was a difference in the rate of immunotherapy treatment change due to toxicity between males and females.

**Methods and Results:**

The Oncology Research Information Exchange Network Avatar Database collected clinical data from 10 United States cancer centers. Of 1035 patients receiving IO, 447 were analyzed, excluding those who did not have documentation noting if a patient changed treatment (*n* = 573). Fifteen patients with unknown or gender‐specific cancer were excluded. All cancer types and stages were included. The primary endpoint was documented treatment change due to toxicity. Four hundred and forty‐seven patients (281 males and 166 females) received IO treatment. The most common cancers treated were kidney, skin, and lung for 99, 84, and 54 patients, respectively. Females had a shorter IO course than males (median 3.7 vs. 5.1 months, respectively, *p* = .02). Fifty‐four patients changed treatment due to toxicity. There was no significant difference between females and males on chi‐square test (11.4% vs. 12.5%, respectively, *p* = 0.75) and multivariable logistic regression (OR 0.924, 95% CI 0.453–1.885, *p* = .827). Significantly more patients with chronic obstructive pulmonary disease (COPD) changed therapy due to toxicity (OR 2.491, 95% CI 1.025–6.054, *p* = .044).

**Conclusion:**

Females received a shorter course of IO than males. However, there was no significant difference in the treatment discontinuation rate due to toxicity between males and females receiving IO. Toxicity‐related treatment change was associated with COPD.

## 1 INTRODUCTION

Immuno‐oncology therapy (IO) has proven efficacious and been approved for the treatment of various advanced solid malignancies such as renal cell carcinoma, urothelial carcinoma, and non‐small cell lung cancer.^1^ Despite their effectiveness, IO has a wide range of toxicities. Commonly reported toxicities of IO include fatigue, colitis, pneumonitis, and various dermatologic, endocrine, cardiac, and neurotoxicities.^1^ While much is known about the side effect profile of IO agents, the sex‐based difference of these toxicities between males and females is largely unknown.

Sex‐related differences in immunity exist, which may alter the efficacy and toxicity profiles of IO in males and females. Females are associated with elevated innate and adaptive immune activity, which likely explains decreased prevalence of some infections and cancers in females and increased prevalence of autoimmune diseases.^2^ Based on these differences, it has been hypothesized that sex differences in IO response exist as well.^3^ However, current data are conflicting on whether sex‐based IO side effect profile differences exist.^4^, ^5^ Unger et al. performed a large retrospective review of patients in clinical trials and showed higher rates of adverse events in females receiving IO. However, Chen et al. reviewed the FDA Adverse Event Reporting System (FAERS) and demonstrated more renal side effects and more severe adverse events in males.^6^, ^7^

We sought to further elucidate the sex‐based difference in IO's toxicity profile by comparing the treatment change rate between males and females receiving IO. We hypothesized that immune‐related adverse events would lead to more frequent treatment changes in females due to their elevated immune response.

## 2 METHODS

**FIGURE 1:**
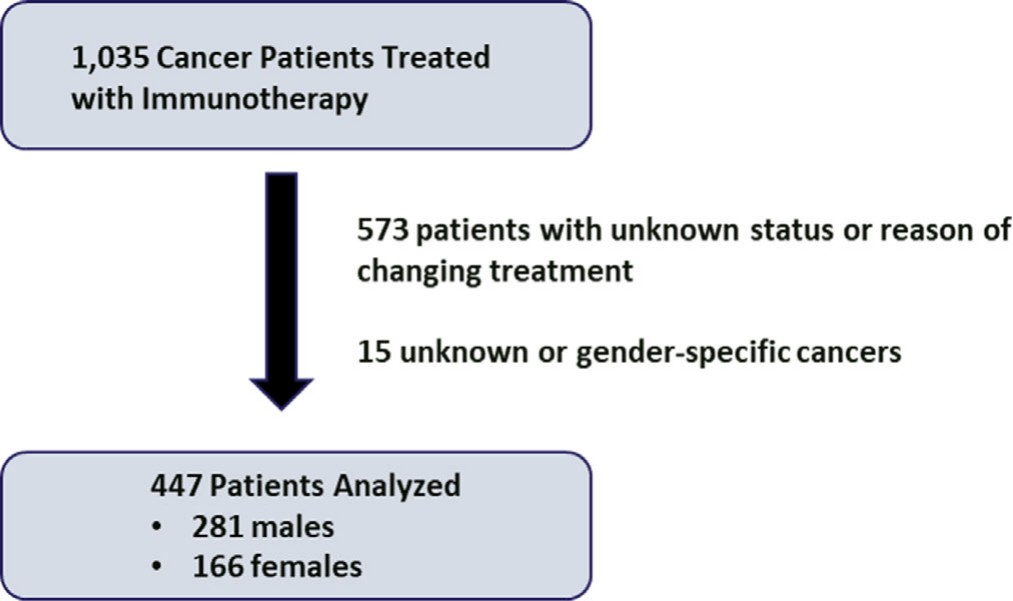
Patient selection.

The Oncology Research Information Exchange Network (ORIEN) Avatar Database is an alliance of cancer centers that allows researchers to share information and expertise. The ORIEN Avatar Database was used to collect a variety of clinical data from 10 different United States cancer centers (Moffitt Cancer Center, The Ohio State University, University of Virginia, University of Southern California, Huntsman Cancer Institute, Stephenson Cancer Center, Holden Comprehensive Cancer Center, Markey Cancer Center, Simon Cancer Center, and the Rutgers Cancer Institute of New Jersey) for patients who received IO. Patient information was de‐identified and was collected from August 2007 to May 2021. The study was approved by the Rutgers University Institutional Review Board. The database included 1035 patients who received IO. Within the database included information regarding if the patient changed treatment which was recorded as “completed treatment”, “no”, “unknown”, “yes”, “yes due to clinical trial”, “yes due to progression”, “yes due to radiation”, and “yes due to toxicity”. Five hundred and seventy‐three patients were excluded if it was unknown if they had changed treatment or if the reason for changing IO treatment was unknown (Figure 1). Fifteen additional patients with an unknown or gender‐specific cancer were excluded. Primary endpoint was documented treatment change due to toxicity. We compared patient characteristics, comorbidities, and length of IO between genders. Cardiovascular comorbidities included history of myocardial infarction, heart failure, ischemic heart disease, hypertension, hyperlipidemia. Endocrine comorbidities included diabetes and hypothyroidism. Thromboembolic comorbidities included previous deep venous thrombosis or pulmonary embolism. Gastrointestinal comorbidities included gastroesophageal reflux disease or hepatic insufficiency. Chronic pain syndromes included chronic pain or neuropathy. Ipilimumab‐nivolumab was considered as combination IO if given at the same time. No other IO combination was identified. Chi squared test was used for non‐parametric categorical variables. Mann Whitney U test was used for non‐parametric continuous variables. Multivariable logistic regression analysis was used to analyze factors associated with changing IO regimens due to toxicity. Multivariable regression analysis was used to analyze factors associated with length of time on IO. Factors included in the multivariable analysis included those that were clinically relevant or had a p‐value less than 0.2 on univariable analysis. In the multivariable linear regression analysis, variables included gender, age, BMI, use of combination immunotherapy, history of COPD, cardiovascular comorbidities, gastrointestinal comorbidities, and chronic pain. In the multivariable logistic regression analysis, variables included gender, age, BMI, history of COPD, and use of combination immunotherapy. Statistical analysis was performed with SPSS version 28.

## 3 RESULTS

Four hundred forty‐seven patients (281 males and 166 females) received IO treatment for cancer. The median age that patients started IO was 59.9 (IQR 50.7–70.4) years for females and 62.9 (IQR 56.8–72.0) years for men. Smoking history was more common in males as current and former smoking status was seen in 10.3% and 41.3% of males and 11.4% and 29.5% of females, respectively (p = 0.01). The most common cancers treated were the kidney (99), skin (84), and lung (54).

Females had a shorter IO course on the Mann‐Whiney *U* test (median 3.7 vs. 5.1 months, respectively, *p* = .02) and multivariable linear regression analysis (*β* −3.87, 95% CI −6.591, −1.149, *p* < 0.01) (Tables 1, 2). Rate of IO treatment change due to toxicity was 12.5% for males (*n* = 35) versus 11.4% for females (*n* = 19) This was not significantly different on chi‐square test (*p* = 0.75) or multivariable logistic regression (OR 0.924, 95% CI 0.453–1.885, *p* = 0.827) (Table 3). More than one different IO was given to 52 patients. IO received included atezolizumab (*n* = 37), avelumab (*n* = 6), cemiplimab (*n* = 2), durvalumab (*n* = 19), ipilimumab (*n* = 30), ipilimumab/nivolumab (*n* = 68), nivolumab (*n* = 151), pembrolizumab (*n* = 190). Patients who changed treatment due to toxicity were given pembrolizumab (*n* = 16), nivolumab (*n* = 14), ipilimumab/nivolumab (*n* = 12), and ipilimumab (*n* = 6), durvalumab (*n* = 3), avelumab (*n* = 2), and atezolizumab (*n* = 1). Out of the 12 who discontinued ipilimumab/nivolumab due to toxicity, four switched to nivolumab alone while eight were not given a second IO. The median time for IO treatment before the change for toxicity was 3.0 months (IQR 1.4–5.9 months). This was also not significantly different between female and males (median time 2.79 months vs. 3.23 months, respectively, *p* = 0.992). There was no difference in changing immunotherapy between females and males due to progression of disease (42.1% vs. 41.3%, respectively, *p* = .85). The strongest association with changing therapy due to toxicity was having chronic obstructive pulmonary disease (COPD) (OR 2.491, 1.025–6.6054; *p* = .004). No significant difference was found in using combination IO (OR 2.122, 0.951–4.738; *p* = .066), being older than 60 years old (OR 0.924, 0.453–1.885; *p* = .827), the female sex (OR 0.924, 0.453–1.885; *p* = .827), or BMI (OR 2.122, 0.951–4.738; *p* = .066) (Table 3).

**TABLE 1:** Baseline characteristics between males and females.

	Male (*n* = 281)	Female (*n* = 166)	*p* value
Age started immunotherapy (IQR, years)	62.9 (56.8–72.0)	59.9 (50.7–70.4)	.01
Median time on immunotherapy (IQR, months)	5.1 (2.1–11.3)	3.7 (1.9–8.1)	.02
BMI (IQR)	27.9 (25.0–31.9)	25.9 (22.6–31.8)	.01
Used a combination Immunotherapy	45 (16%)	23 (13.9%)	.54
Smoking status			.01
Current	29 (10.3%)	19 (11.4%)	
Former	116 (41.3%)	49 (29.5%)	
Cardiovascular comorbidity	184 (65.5%)	91 (54.8%)	.03
Endocrine comorbidity	82 (29.2%)	55 (33.1%)	.38
COPD	19 (6.8%)	21 (12.7%)	.38
Renal failure	19 (6.8%)	7 (4.2%)	.27
Thromboembolic comorbidity	14 (5.0%)	10 (6.0%)	.64
Gastrointestinal comorbidity	54 (19.2%)	45 (27.1%)	.05
Chronic pain syndrome	98 (34.9%)	54 (32.5%)	.61

**TABLE 2:** Linear regression analysis of risk factors associated with length of time on immunotherapy.

	Univariable			Multivariable		
	*β*	95% CI	*p* value	*β*	95% CI	*p* value
Female gender	−1.595	−4.014, 0.824	.195	−3.87	−6.591, −1.149	.005
Age ≥ 60	−0.176	−2.552, 2.199	.884	−0.62	−3.37, 2.133	.658
BMI ≥ 25	−2.05	−4.763, 0.664	.138	−2.49	−5.282, 0.311	.081
Cardiovascular comorbidity	−1.651	−4.066, 0.764	.18	−0.55	−3.44, 2.346	.71
COPD	−2.629	−6.526, 1.269	.186	−1.78	−5.849, 2.290	.39
Used combination immunotherapy	0.351	−2.754, 3.456	.824	−0.85	−4.307, 2.605	.628
Chronic pain syndrome	−2.892	−5.348, −0.436	.021	−2.5	−5.094, 0.100	.059
Gastrointestinal comorbidity	0.113	−4.954, 0.527	.113	−1.39	−4.327, 1.551	.353

**TABLE 3:** Logistic regression analysis of risk factors associated with changing immunotherapy due to toxicity.

	Univariable			Multivariable		
	OR	95% CI	*p* value	OR	95% CI	*p* value
Female	0.908	0.501–1.647	.752	0.924	0.453–1.885	.827
Age ≥ 60	1.241	0.688–2.240	.473	1.409	0.699–2.842	.338
BMI ≥ 25	0.838	0.426–1.648	.608	0.76	0.374–1.544	.448
COPD	2.335	1.045–5.220	.039	2.491	1.025–6.054	.044
Combo IO	1.719	0.853–3.466	.13	2.122	0.951–4.738	.066

## 4 DISCUSSION

Our study showed no significant difference between sex for treatment change due to IO toxicity effects. The strongest association with treatment change due to toxicity was COPD. The median time of IO therapy before treatment change due to toxicity was 3.0 (IQR 1.4–5.9) months. There was no significant difference seen in treatment change due to toxicity when using combination therapy.

Since females have a stronger innate and adaptive immune response, we hypothesized that there would be an enhanced response to IO which could lead to increased adverse events.^3^ However, we found that there was no significant difference in treatment change due to toxicity between sex. Immunologic sex differences have been seen before in the setting of vaccine response and autoimmune diseases. For instance, females have been shown to develop more significant responses to vaccines where one trial demonstrated it only took half the flu vaccine dose to developed a similar antibody response in men with the full vaccine dose.^8^ However, flu vaccine adverse events have also been reported to be up to two to three times more frequent in females.^3^, ^8^, ^9^ Autoimmune diseases (e.g., rheumatoid arthritis [RA]) are also three times more prevalent in females, and females frequently have more severe and earlier‐onset autoimmune diseases.^3^ Additionally, sex‐related differences in biologic treatments for RA have been seen with lower remission rates of RA in females compared to males when taking TNF inhibitor therapies.^10^, ^11^, ^12^, ^13^

There are several reasons why immunologic differences may exist between sex. One theory is that women have an increased immune response due to higher expression of immune related genes from a second X chromosome. While one X chromosome is supposed to be inactivated, several genes termed “escape of X‐chromosome inactivation” (EXIT) genes can escape X‐inactivation and lead to increased transcription of immune response genes.^14^, ^15^ A second theory is related to the different amount of sex hormones produced between genders as they can also influence immune cell function. Estrogen can lead to increased immune response including activation of B‐cell function, CD4 T cell activation, increase intracellular PD1 expression and increasing various cytokine production.^16^, ^17^ Meanwhile, increased androgen signaling may play a role in impaired CD8^+^ T cells.^18^

The role of gender effects is also an understudied topic that can lead to differences in IO response; gender is often intertwined with one's sex and not teased out separately but may play an additional or separate role in outcomes. Sex is thought to have its effect from the biological standpoint, where gender plays a role in its outcomes from a social context.^16^ Gender‐related factors include psychosocial, behavior, social, and cultural differences.^3^ Several gender‐related risk factors have been described which include disparity in exposure to levels of tobacco, alcohol, environmental exposures, body weight, dietary patterns, and physical activity.^16^

Social roles, health‐seeking behavior, and gender bias in drug prescribing behaviors and toxicity assessment by practitioners can also affect outcomes for respective genders. Some medication adverse effects may lead to hair loss or weight gain, and due to cultural norms, a gender disparity in medication adherence may exist due to the effect on one's self‐image.^3^, ^19^ Also, men are less likely to report health effects perceived as less severe, whereas it is deemed more socially acceptable for women to seek healthcare.^3^ There is a large variety of sex and gender factors that can influence rates of adverse events or explain why a significant difference in length of IO treatment was seen in our study's female population.

While there are many potential sex and gender‐related factors that can affect IO outcomes, there has been a paucity of literature regarding sex‐related differences in immune‐related adverse events (irAE), and those which have been performed have mixed results. For instance, Duma et al. evaluated sex differences of irAEs for anti‐PD‐1 therapy and demonstrated increased irAEs in females compared to males in 231 non‐small cell lung cancer patients (48% vs 31%, respectively, *p* = .008) and 245 melanoma patients (67% pre‐menopausal women vs. 60% post‐menopausal women vs. 46% men, *p* = .04).^20^ Additionally, they showed no significant difference in discontinuation due to toxicity between genders for melanoma (23% pre‐menopausal women vs. 15% post‐menopausal women vs. 12% men, *p* = .28), but a significant difference in non‐small cell lung cancer (17% women vs. 7% men, *p* = .04). Meanwhile, Cortellini et al. demonstrated no significant difference in irAEs between sex for 1010 non‐small cell lung cancer patients being treated with pembolizumab, and 9.1% of patients discontinued treatment due to adverse effects.^21^ Interestingly, Kartolo et al. showed males had increased irAEs compared to females when studying 78 patients receiving IO.^22^ Recently, two studies with larger sample sizes were published, but also had mixed outcomes. Chen et al. reviewed irAEs in the FAERS database and identified 11 097 reports from females and 19 245 reports from males.^7^ In the analysis, males had increased renal toxicity and more severe irAEs compared to females. Unger et al. evaluated differences in adverse effects in a variety of cancer treatments including IO for patients in a clinical trial. Symptomatic adverse effects were greater for females compared to males receiving immune checkpoint inhibitors (*n* = 877; 19.6% vs. 13.0% respectively; OR 1.54; *p* = .03) and immune system modulators (*n* = 1243; 44.1% vs. 33.6% respectively; OR = 1.62; *p* < .001).^6^ In our study, we demonstrated that there was no sex differences IO treatment change due to adverse effects. While these results are mixed, it is possible that differences exist based on a variety of factors such as genetics, type of IO, and cancer type.^23^ As each of these studies were performed retrospectively, future studies should prospectively collect data and carefully document sex, gender, type of adverse event grade of adverse event, and molecular profiling data.

## 5 LIMITATIONS

Our study had several limitations. First, it was a retrospective analysis. Second, the sample size is relatively small with a variety of IO being used for different cancers and stages. Third, it is possible that treatment duration between sexes were significantly different due to the difference of efficacy between sexes. While treatment efficacy was not captured in our analysis, we demonstrated that there was no significant difference in changing IO due to progression between sex. Fourth, specific toxicities were not well recorded. The ORIEN database records only a few toxicities if they are greater than a grade 2 adverse event including neurologic, thromboembolic, liver, renal toxicities, and fatigue. However, out of the 54 patients that change treatment regimen due to toxicity, only 3 had a documented adverse event which were all fatigue. Furthermore, symptoms from a patient's underlying disease and comorbidities can contribute to toxicity; however, we are unable to assess this from the database. Fifth, we were unable to assess the usage of IO + chemo or targeted therapy which may also affect toxicity. Likely, this database did not show a significant difference in adverse events compared to men due to the limitations of the database and the small sample size. Future studies should aim to document specific side effects and their grade of toxicity given that IO can cause a wide range of toxicities with different severity. Despite the small sample size and limited follow up time, the ORIEN database offers the ability to evaluate patients with a variety of cancers receiving different IO and therefore may better identify sex differences in IO outcomes and reasons for discontinuation as patients accrue.

## 6 CONCLUSION

Females received a significantly shorter course of IO than males in the ORIEN Avatar database. There was no significant difference in the treatment discontinuation rate due to toxicity between males and females receiving IO. Toxicity‐related treatment change was associated with COPD. The database was limited in its ability to analyze the type or severity of AEs due to IO. Studies with larger sample sizes with more granular data (i.e., type of adverse effects) are needed to determine if a difference between sex, gender, and IO toxicity exists.

## AUTHOR CONTRIBUTIONS

**Kevin Chua:** Conceptualization (equal); data curation (lead); formal analysis (lead); investigation (lead); methodology (lead); resources (equal); visualization (equal); writing – original draft (equal); writing – review and editing (equal). **Shane Kronstedt:** Investigation (equal); visualization (equal); writing – original draft (equal); writing – review and editing (equal). **Alain Kaldany:** Conceptualization (equal); data curation (equal); investigation (equal); methodology (equal); writing – original draft (equal); writing – review and editing (equal). **Arnav Srivastava:** Investigation (equal); methodology (equal); supervision (equal); validation (equal); visualization (equal); writing – original draft (equal); writing – review and editing (equal). **Sai Krishnaraya Doppalapudi:** Conceptualization (equal); investigation (equal); methodology (equal); visualization (equal); writing – original draft (equal); writing – review and editing (equal). **Hao Liu:** Formal analysis (supporting); methodology (supporting); supervision (equal). **Ahmad A. Tarhini:** Project administration (equal); supervision (equal); writing – review and editing (equal). **Elizabeth Gaughan:** Project administration (equal); supervision (equal); writing – review and editing (equal). **Siwen Hu‐Lieskovan:** Project administration (equal); supervision (equal); writing – review and editing (equal). **Raid Aljumaily:** Project administration (equal); supervision (equal); writing – review and editing (equal). **Kenneth Nepple:** Project administration (equal); supervision (equal); writing – review and editing (equal). **Bryan Schneider:** Project administration (equal); supervision (equal); writing – review and editing (equal). **Joshua Sterling:** Conceptualization (equal); formal analysis (equal); investigation (equal); methodology (equal); project administration (equal); resources (equal); supervision (equal); visualization (equal); writing – original draft (equal); writing – review and editing (equal). **Eric Singer:** Conceptualization (equal); formal analysis (equal); investigation (equal); methodology (equal); project administration (equal); resources (equal); supervision (equal); writing – original draft (equal); writing – review and editing (equal).

## FUNDING INFORMATION

This work is supported by a grant from the National Cancer Institute (P30CA072720).

## CONFLICT OF INTEREST STATEMENT

Ahmad Tarhini contracted research grants with institution from Bristol Myers Squib, Genentech‐Roche, Regeneron, Sanofi‐Genzyme, Nektar, Clinigen, Merck, Acrotech, Pfizer, Checkmate, OncoSec, Scholar Rock, InflaRx GmbH, Agenus; personal consultant/advisory board fees from Bristol Myers Squibb, Merck, Easai, Instil Bio, Clinigin, Regeneron, Sanofi‐Genzyme, Novartis, Partner Therapeutics, Genentech/Roche, BioNTech, Concert AI, AstraZeneca outside the submitted work. Margaret Gatti‐Mays: Advisory board for SeaGen and GE Healthcare. Eric A. Singer provided research support‐Astellas/Medivation; Advisory board/consulting‐Merck, Johnson & Johnson, Vyriad, Aura Biosciences. All other authors declare no conflict of interest.

## ETHICS STATEMENT

We utilized real‐world clinical data collected under the Total Cancer Care Protocol (NCT03977402) and Avatar® project within the Oncology Research Information Exchange Network (ORIEN) of 10 cancer centers to which all included subjects provided an IRB‐approved written informed consent at their participating institutions.

## Data Availability

Data sharing is not applicable to this article as no new data were created or analyzed in this study.
